# The mediation role of work–family conflict in the effect of workplace violence on job satisfaction and intention to leave: a study on health care workers in Turkey

**DOI:** 10.3389/fpsyg.2024.1322503

**Published:** 2024-04-08

**Authors:** Abdulhamit Tutan, Özgür Kökalan

**Affiliations:** ^1^Graduate Education Institute, Istanbul Sabahattin Zaim University, Istanbul, Türkiye; ^2^Department of Business Administration, Istanbul Sabahattin Zaim University, Istanbul, Türkiye

**Keywords:** workplace violence, job satisfaction, work–family conflict, intention to leave, health care

## Abstract

**Background:**

This study aims to determine how workplace violence experienced by healthcare workers in Turkey affects their job satisfaction and intention to leave. It also examines the mediating role of employees’ work-family conflict between these effects.

**Methods:**

The PROCESS method was used in the study. The research was conducted on 595 health workers in three public hospital affiliated with the Istanbul Provincial Health Directorate. The convenience sampling method was used in the selection of the participants.

**Results:**

As a result of the analysis, it was determined that there is a positive, significant, moderate (*R* = 0.35, *p* < 0.01) relationship between workplace violence and work-family conflict, and a negative, significant and weak relationship between workplace violence and job satisfaction (*R* = −0.27, *p* < 0.01), there is a positive, significant, and weak (*R* = 0.26, *p* < 0.01) relationship between workplace violence and intention to leave. In addition, there is a negative, significant, and weak (*R* = −0.27, *p* < 0.01) relationship between work-family conflict and job satisfaction, and a positive, significant, and weak (*R* = 0.28, *p* < 0.01) relationship between work-family conflict and intention to leave. Workplace violence had significant and negative effects on the employees’ job satisfaction and significant and positive effects on the intention to leave and work-family conflicts.

**Discussion:**

As a result of the mediating variable analysis, it was determined that work-family conflict has a partial mediator role in the relationship between workplace violence, job satisfaction, and intention to leave. The results are very important, especially for managers working in the healthcare sector. Reducing workplace violence against healthcare personnel will contribute to increasing productivity in the sector and providing better quality service to the healthcare sector.

## 1 Introduction

Workplace Violence (WPV) is a global public health problem and poses a serious threat. It is accepted that the risk of workplace violence among individuals working in the health sector due to their working conditions is greater than that of individuals working in all other sectors (Usluoğulları and Turan, 2023). In the report published by the World Health Organization (WHO) and the International Labor Organization (ILO) in 2002, it was reported that “25% of the violence in all sectors occurs in the health sector, and more than 50% of the healthcare workers are exposed to violence at any time they practice their profession” (Kılıç and Çolak, 2020). According to the same report, when we look at the types of violence experienced by workers in the health sector, 10–23% have encountered psychological violence, 0.8–2.7% ethnic violence, 27–67% verbal violence, 3–17% physical violence, and 0.7–0.8% have been subjected to sexual violence (Kılıç and Çolak, 2020). According to the results of the study conducted by the Turkish Health and Social Workers Union, 87% of the health personnel in Turkey stated that they had been subjected to verbal, psychological, or physical violence at least once during their professional life. The rate of those who say that they have been subjected to verbal, psychological, or physical violence at least once in the last 12 months in the health sector in Turkey is 82% (Aydın, 2018).

The source of workplace violence in the health sector can be the patient, the patient’s relative, and the health worker. It undeniably affects health workers in terms of physical and mental health (Giorgi et al., 2020). WHO has addressed this effect in physical and psychological dimensions. The exposure of healthcare professionals to violence affects not only the health of the relevant employee, but also many organizational factors, such as the employee’s job satisfaction, work-family life, and commitment to that institution (Esen and Aykal, 2020). Therefore, the increase in work–family conflict (WFC) and job dissatisfaction among health workers due to exposure to workplace violence, and the resulting increased intention to leave, negatively affects not only the health worker as an individual but also the quality of the health profession and indirectly the service quality of the sector will affect.

This study aimed to determine how workplace violence experienced by employees in the Turkish health sector affects their job satisfaction and intention to leave and the mediating role of employees’ WFC between these effects. Determination of job satisfaction and WFC levels of health workers as a result of workplace violence and their intention to leave the job; will ensure that the institutions have a motivating potential to produce policies in this area and to make the necessary improvement. In addition, the database to be created in order to identify the problems in the health sector will enable the classification of these problems and their analysis for targeted interventions. Thus, this field will contribute to raising awareness and developing social consciousness. At the same time, it will also form the basis for future studies.

In addition, it is thought that determining the workplace violence, WFC conflict, and job satisfaction levels of health workers and their intention to leave the job will contribute to the prevention of both material and moral losses, and the productivity and development of the sector. Although there are many studies on workplace violence in the health sector in the literature, no study examines the effects of these concepts in a holistic way in the Turkish health sector. For this reason, the study will contribute to the literature.

### 1.1 Workplace violence

Many definitions have been made in studies on workplace violence. Workplace violence in the most general sense, has been expressed as “excessive mood, intensity, the harshness of a case, rude and harsh behavior, abuse of body power, activities that harm the individual and society” (Akkaş and Uyanık, 2016). The concept of violence faced by healthcare professionals is defined as “coming from the patient, patient relatives or any other individual, which poses a risk to the healthcare worker; threatening behavior, verbal threat, economic abuse, physical assault, and sexual assault” (Cenger et al., 2018).

When we look at the types of violence that are exposed or realized in the workplace, we can see that it covers physical violence, psychological violence, abuse, mobbing, harassment, sexual harassment, threat, and racial harassment (World Health Organization, 2002). According to the bibliometric analysis study on workplace violence, it is stated that the most common types of violence are physical violence, verbal violence, and sexual harassment (Seyran, 2021).

Working in the health sector is much riskier than other sectors in terms of exposure to violence. It is seen that this risk is 16 times higher in various studies conducted in the health sector. Within the health sector, 80% of all violent incidents experienced by healthcare professionals are patient or patient’s relative and service-related violence incidents (Cebrino and Cruz, 2020; Ayyıldız, 2021).

In a study examining on workplace violence, it was found that health workers were exposed to violence at a rate of 62% in the last year, and this rate included non-physical violence (verbal harassment, threats, and sexual harassment) by 42.5% and physical violence by 24.4% indicated to include violence. In the same study, especially in Asian and North American countries, Nurses and doctors working in Psychiatry and Emergency Departments have been reported to have a high rate of exposure to violence (Liu et al., 2019).

In another study, article on emergency room doctors and nurses were examined, it was stated that 72% of health workers were exposed to verbal violence and 18% to physical violence, of which approximately 37% were doctors, 56% nurses and approximately 7% other health workers (Aljohani et al., 2021).

As a result of the various studies show that the violence exposed at the workplace affects the motivation and performance of the employee, reducing his potential productivity and creativity. This situation causes depression, anxiety disorder, social alienation, job incompatibility, and work alienation in the employee, which leads to negative consequences such as job dissatisfaction, decreased organizational commitment, WFC, and intention to leave (Duan et al., 2019; Antão et al., 2020).

### 1.2 Job satisfaction

The concept is generally in the literature; it has also been expressed as “the person’s satisfaction with his/her job” or “positive feelings toward his/her job” (Roelen et al., 2008). Job satisfaction is an employee’s view of his/her job in general (Robbins, 1991). Job satisfaction, one of the most important factors affecting the behavior of employees in all organizations, is generally expressed as a reflection of the employee’s satisfaction with the job description (Tutan and Öztürk, 2020). The opportunities offered by the enterprises can have an important role in meeting the wishes and needs of the employees and ensuring job satisfaction. The factor that creates job satisfaction is grouped under two groups as factors related to the person and the job. While explaining the employee’s personality, work experience, and social life as factors related to the person, The appearance and degree of difficulty of the job, the internal characteristics of the job, the salary, the opportunity for advancement, the reward, the human relations in the enterprise, the social appearance of the enterprise, the working conditions and job security are expressed as the factors related to the job (Demirbaş et al., 2022). In addition, individual factors affecting job satisfaction are listed as demographic characteristics, age, gender, education level, working time, and marital status (Günday et al., 2022).

There are different evaluations regarding the factors affecting job satisfaction in the literature, but it is understood that the most common classification is “individual and organizational factors” (Nguyen, 2020).

The study conducted for all occupational groups, it was determined that health workers are the occupational group with the lowest job satisfaction (Özer and Bölüktaş, 2022). According to the results of study on nurses, a negative and moderate relationship was observed between mobbing and the job satisfaction of nurses (Kurnaz and Oğuzhan, 2021).

### 1.3 Work–family conflict (WFC)

The family, one of the most important elements of social life, should consist of individuals who are in well psychological and physical health. This is possible when individuals balance both their work and family life (Bojan et al., 2020). However, positive or negative situations in work or family life can affect the others and even lead to conflicts, and for these reasons balance is not achieved. Greenhaus and Beutell explained that as the incompatibility between different roles undertaken in work and family life increases, work–family conflict also increases (Adisa and Gbadamosi, 2021). Work-family or family–work conflict arises as a result of these two concepts being affected by each other (Erer, 2021).

It is stated that work–family conflict consists of three dimensions (time, strain, behavior) (Chandler, 2021). Time-based conflict is when the time to be allocated for one role interferes with the time to be allocated for another role (İskender and Yaylı, 2021). Work-related causes of this type of conflict include long working hours, frequency of overwork, shift system and irregularity, and inflexible work schedules (Silva and Dissanayake, 2017). Strain-based conflict is the inability to fulfill responsibilities in the other role due to unrest (weakness, anxiety, depression, tension, fatigue, etc.) arising in one of the roles. Factors such as role ambiguity, excessive workload, high physical and psychological work demands, frequent changes in the work environment, poor communication at the workplace, and work commitment are among the causes of Strain-based work–family conflict (Yang et al., 2022) Behavior-based conflict is when the attitude specific to one role is not compatible with the attitude in the other role (Ameti and Abaz, 2023). Behavior-based conflict occurs when the person cannot change his/her behaviors according to his/her roles.

According to the study result, WFC affects job satisfaction, organizational commitment, intention to leave, absenteeism, job performance, career satisfaction, and career success, it has an effect on life satisfaction, marital satisfaction, family performance, and leisure satisfaction in non-work results (Allen et al., 2000).

In studies on healthcare workers, it has been determined that WFC has a negative and significant effect on job satisfaction and organizational commitment levels, and a positive and significant effect on burnout (Mutlu et al., 2021).

### 1.4 Intention to leave

Employees’ intention to leave is a destructive and active action when they are not satisfied with their working conditions (Uludağ, 2019). According to another definition, it is expressed as the thought of leaving their jobs due to the employee’s dissatisfaction with the institution’s working conditions (Özcan et al., 2012).

In studies conducted on healthcare workers, it has been determined that the intention to leave work is positively related to WFC and negatively and significantly related to job satisfaction, and these two concepts have a negative effect (Blanco-Donoso et al., 2021; Tariq et al., 2021).

In a study conducted on healthcare workers in Switzerland, it was found that workplace violence and discriminatory behaviors in the work environment lead to employees intending to leave their jobs, resulting in job changes or career terminations (Hämmig, 2023).

### 1.5 Job satisfaction with exposure to violence, work–family conflict, and intentions to leave the work of healthcare professionals

Violence, which has become a part of social life, is frequently experienced in health institutions. Workplace violence, which has different negative results in institutional and individual dimensions, does not only have consequences such as depression and anger but also affects internal communication and damages the organizational climate. On the other hand, all these factors decrease the job satisfaction of the institution’s employees, indirectly cause WFC, ultimately increase the tendency to leave the job, and increase the employee turnover rate (Ünlüsoy et al., 2023). When evaluated within this framework, there is an interaction between workplace violence, WFC, job satisfaction, and turnover tendency. Increasing exposure to violence in institutions reduces job satisfaction, thus increasing WFC and ultimately increasing the intention to leave (Milet and Yanık, 2017). In other words, as the frequency and duration of exposure to violence increases, a vicious circle occurs, the employee’s organizational commitment, whose intention to leave the job increases, decreases, creates more conflict and reduces job satisfaction (Ersoy, 2020). The employee turnover rate among healthcare workers is very high. This situation may prevent health professionals from reaching professional status. For this reason, workplace violence, WFC, job satisfaction, and turnover intention in healthcare workers are becoming increasingly important when considering today’s world.

A study stated that while workplace violence had a direct and significant effect on job satisfaction and intention to leave, job satisfaction was negatively related to intention to leave (Zhao et al., 2018). In another study, it was found that workplace violence has a positive relationship with intention to leave and burnout, while also having a negative relationship with job satisfaction (Duan et al., 2019). In another study conducted with healthcare workers, it was reported that intention to leave is negatively related to job satisfaction and organizational commitment, while burnout has a positive significant relationship with intention to leave (Liu et al., 2019). It has been stated that workplace violence experienced by healthcare workers significantly negatively impacts their job satisfaction and success at work, and this seriously affects their intention to leave the job. In addition, it has been stated that while job satisfaction is a full mediator between workplace violence and job satisfaction, and partially mediates the intention to leave (Zhao et al., 2018). In another study, it was stated that workplace violence experienced by nurses had a positive effect on and intention to quit (Cho and Lee, 2017). In another study, it was observed that workplace violence experienced by healthcare workers affects job satisfaction, the intention to leave the job, and burnout. Additionally, the same study indicated that workplace violence and job satisfaction mediate the relationship between burnout and the intention to leave the job (Gedik et al., 2023).

**Figure 1:**
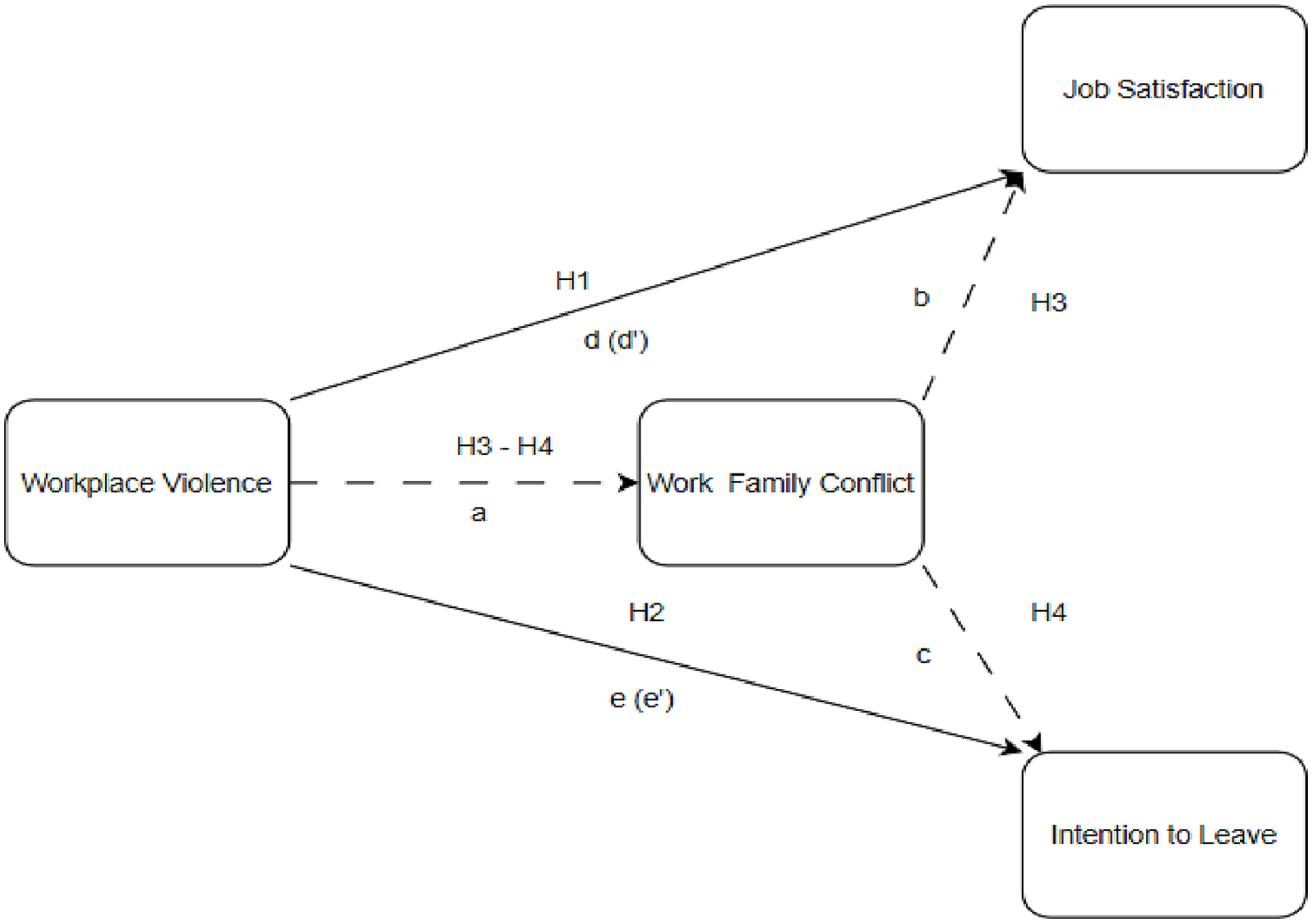
Theoretical model.

This study was conducted to determine how workplace violence experienced by health sector workers affects their job satisfaction and intention to leave, and whether work–family conflict plays a mediating role in this interaction. When all theoretical studies (Figure 1) are examined, the hypotheses and research model (Figure 1) to be used in this study are formed as follows:

*H1*: Workplace violence experienced by healthcare professionals significantly affects their job satisfaction.

*H2*: Workplace violence experienced by healthcare professionals significantly affects their intention to leave.

*H3*: Work–family conflict mediates the interaction between workplace violence experienced by health workers and job satisfaction.

*H4*: Work–family conflict mediates the interaction between workplace violence experienced by healthcare professionals and their intention to leave.

## 2 Methods

### 2.1 Design and sample

The research was conducted face to face on 595 healthcare professionals working in 3 public hospitals in İstanbul. The data obtained was digitized and transferred to the SPSS application. The convenience sampling method was used in the selection of the participants. After the ethical approval was obtained, the data collection phase was carried out over 4 months, from May to October 2022. To reduce the common method bias problem, the questionnaire was distributed two times with a 45-day break (Podsakoff et al., 2012). It is stated that at least 200 samples should be used to minimize the calculation errors in mediator variable analysis (Kline, 2011). Within the framework of all references, it is seen that the number of samples used in the research is sufficient for mediator variable analysis.

### 2.2 Instruments and measures

The questionnaire used in the research consists of five parts. The first part consists of 7 questions aiming to determine the socio-demographic characteristics of the participants. The second part is the workplace violence scale consisting of 9 questions. The work–family conflict scale in the next section consists of 5 questions. The fourth part is a job satisfaction scale consisting of 5 questions. In the last part, there is the intention to quit job scale consisting of 3 questions. The data were analyzed with AMOS 25 and SPSS 25 programs.

Workplace Violence Scale was developed by Chen et al. (2004) and adapted into Turkish by Tutan and Kökalan (2023). The scale consists of 9 questions and three sub-dimensions called “Verbal Violence,” “Physical Violence” and “Sexual Violence.” In the scale, a 4-point Likert-type scale was used, which was expressed as “Never,” “1 Times,” “2–3 Times,” and “>3 More Times.” Example items included “In the last 12 months, I have been exposed to scolding, insulting, humiliating, humiliating or damaging the dignity of the individual (face-to-face, telephone, letters or brochures, micro-network, etc.),” “In the last 12 months, verbal I have been subjected to sexual assault and provocation (verbal abuse or acts of harassment, harassment through the display of genitals, etc.). High scores indicated high levels of Workplace Violence.

Work–Family Conflict Scale was developed by Netemeyer et al (1996) and adapted into Turkish by Apaydın (2004). The scale consists of two dimensions called “Work–Family Conflict” and “Family–Work Conflict” and 10 statements. Of these dimensions, only the “Work–Family Conflict” dimension was included in the study. Scale statements were measured with a 5-point Likert-type scale, which was expressed as “Strongly Disagree,” “Agree,” “I am undecided,” “Agree” and “Strongly Agree.” Example items included “The stress of my job makes it difficult for me to fulfill my duties toward my family,” “The time I spend on my job makes it difficult for me to fulfill my responsibilities toward my family.” High scores indicated high levels of WFC.

Job Satisfaction Scale was developed Brayfield and Rothe developed the scale Brayfield and Rothe (1951), shortened by Judge et al (1998), and adapted into Turkish by Başol and Çömlekçi (2020). Scale statements were measured with a 5-point Likert-type scale, which was expressed as “Strongly Disagree,” “Agree,” “I am undecided,” “Agree” and “Strongly Agree.” Example items included “I find happiness in my job,” “I do my job with love.” High scores indicated high levels of Job Satisfaction.

The Intention to Leave Scale was developed by Rosin and Korabik (1991) and adapted into Turkish by Atay (2012). The scale consists of one dimension and three statements. Scale statements were measured with a 5-point Likert-type scale, which was expressed as “Strongly Disagree,” “Agree,” “I am undecided,” “Agree” and “Strongly Agree.” Example items included “I would quit my job if I had the opportunity,” “I have recently started to think about quitting my job more often.” High scores indicated high levels of Intention to Leave.

### 2.3 Data analyzes

The data analysis of the research was carried out with quantitative methods. Before proceeding to the mediator variable analysis, Confirmatory Factor Analysis (CFA) was applied separately for the three scales. According to the results obtained in the CFA results, it was seen that all the scales were at a sufficient level. In the next step, the reliability of the three scales was evaluated with the Cronbach Alpha coefficient. It is seen that the Cronbach Alpha results of the scales vary between 0.78 and 0.91. The reliability values of all scales are higher than 0.70. This shows that the scales are reliable enough to analyze (Meydan and Şeşen, 2001). The index results of the scales obtained as a result of CFA and Cronbach’s Alpha coefficients are briefly summarized in Table 1.

**Table 1:** Fit index results of scales and Cronbach alpha coefficients.

	χ^2^/df	CFI	AGFI	NFI	GFI	RMSEA	Cronbach alfa
Minimum Threshold Values	≤5	0.90	0.80	0.85	0.85	≤0.10	≥0.70
Workplace Violence Scale	4.74	0.92	0.86	0.88	0.94	0.09	0.81
Work–Family Conflict Scale	4.56	0.98	0.92	0.98	0.97	0.08	0.91
Job Satisfaction Scale	3.88	0.99	0.96	0.99	0.99	0.05	0.90
Intention to Leave the Job Scale	3.95	0.99	0.96	0.98	0.99	0.05	0.88

χ^2^/df, Chi-Square/degrees of freedom; RMSEA, root mean square error of approximation; NFI, normed fit index; GFI, goodness of fit index; AGFI, adjusted goodness of fit index; CFI, comparative fit index.

### 2.4 Ethical considerations

Within the scope of the study, firstly, the necessary approval was obtained from the İstanbul Sabahattin Zaim University Ethics Commission with the number 2022/04 and the number E-20292139-050.01.04-27224 on 29.04.2022, then from the Istanbul Provincial Health Directorate on 22.09.2022 to be used in public hospitals. Ethical approval was obtained with the number/12411 and the number E-15916306-604.01.01. At the same time, before filling out the questionnaire, the participants were informed about the purpose of the study, and their consent was obtained about whether or not to participate in the study.

## 3 Results

### 3.1 Demographic results

Information about the socio-demographic characteristics of the participants is summarized in Table 2.

**Table 2:** Socio-demographical characteristics of the participants.

		Frequency	Percent (%)	Valid percent (%)	Cumulative percent (%)
Gender	Male	185	31.1	31.1	31.1
	Women	410	68.9	68.9	100.0
Marital status	Married	359	60.3	60.3	60.3
	Single	236	39.7	39.7	100.0
Age	30 under	257	43.2	43.2	43.2
	31–40 age	212	35.6	35.6	78.8
	41–50 age	83	13.9	13.9	92.8
	50 over	43	7.2	7.2	100.0
Education level	High school	54	9.1	9.1	9.1
	Associate degree	46	7.7	7.7	16.8
	Bachelor degree	240	40.3	40.3	57.1
	Graduate	255	42.9	42.9	100.0
Work experience	Less than 1 year	44	7.4	7.4	7.4
	1–5 years	227	38.2	38.2	45.5
	6–10 years	149	25.0	25.0	70.6
	Over 10 years	175	29.4	29.4	100.0
	Doctor	315	52.9	52.9	52.9
Profession group	Nurse	105	17.6	17.6	70.6
	Health technician	65	10.9	10.9	81.5
	Medical secretary	30	5.0	5.0	86.6
	Auxiliary staff	27	4.5	4.5	91.1
	Other	53	8.9	8.9	100.0
	Internal services	113	19.0	19.0	19.0
Worked unit	Surgical services	154	25.9	25.9	44.9
	Intensive care	52	8.7	8.7	53.6
	Operating room	21	3.5	3.5	57.1
	Emergency	59	9.9	9.9	67.1
	Policlinic	85	14.3	14.3	81.3
	Other	111	18.7	18.7	100.0
	Total	595	100.0	100.0	

In Table 2, it is seen that 410 (68.9%) of the participants are female, 185 (31.1%) are male, 60.3% are married, and 39.7% are single. 43.2% of the participants are under 30 years old, 35.6% are 30–40 years old, 21.1% are over 40 years old, 16.8% are at least an associate degree, and 40.3% are undergraduate and graduate students. It is seen that 42.9% of them have a master’s degree. 45.6% of the participants have at least 5 years of work experience, 54.4% have at least 6 years or more work experience, 52.9% are doctors, 17.6% are nurses, 10.9% These are health technicians, 5% are medical secretaries, 4.5% are auxiliary personnel, and 8.9% belong to other professions, 19% are in internal services, 25.9% are in surgical services, 8% are in 0.7% of their work in the intensive care unit, 3.5% in the operating room, 9.9% in the emergency room, 14.3% in the polyclinic and 18.7% in other units.

### 3.2 Key statistics on workplace violence, work–family conflict, job satisfaction, and intention to leave

In this part of the study, the arithmetic means, standard deviation, skewness, and kurtosis values of the variables of workplace violence, WFC, job satisfaction, and intention to leave are calculated, and the obtained values are briefly summarized in Table 3.

**Table 3:** Key statistics on workplace violence, work–family conflict, job satisfaction, and intention to leave.

	*N*	Arithmetic mean	Standard deviation	Skewness	Kurtosis
Workplace violence	595	1.4256	0.42151	1.231	1.829
Verbal violence	595	2.0588	0.94899	0.600	−0.688
Physical violence	595	1.1188	0.83458	1.490	1.218
Sexual violence	595	1.0992	0.71954	1.032	1.656
Work–family conflict	595	3.7442	1.04967	−0.852	0.088
Job satisfaction	595	3.1724	0.93047	−0.445	−0.232
Intention to leave	595	2.7605	1.09943	0.305	−0.658

As can be seen in Table 3, the workplace violence levels of the participants (M: 1.42; Std: 0.42) are low, WFC (M: 3.74; Std: 1.05) is high, job satisfaction (M: 3.17; Std: 0.93), while their intention to leave (M: 2.76; Std: 1.1) is moderate. When the skewness and kurtosis values of all variables are examined, it is seen that the data used in the research are normally distributed (Meydan and Şeşen, 2001).

### 3.3 Correlation analysis between the variables of workplace violence, work–family conflict, job satisfaction, and intention to leave

In this part of the research, the relationships between workplace violence, WFC, job satisfaction, and intention to leave were analyzed with the Pearson Correlation test. The obtained results are briefly summarized in Table 4.

**Table 4:** Results of correlation analysis between variables of workplace violence, work–family conflict, job satisfaction and intention to leave.

		1	2	3	4	5	6	7
1	Workplace violence	1						
2	Verbal violence	0.92^**^	1					
3	Physical violence	0.65^**^	0.40^**^	1				
4	Sexual violence	0.53^**^	0.25^**^	0.34^**^	1			
5	Work–family conflict	0.35^**^	0.35^**^	0.12^**^	0.10^**^	1		
6	Job satisfaction	−0.27^**^	−0.27^**^	−0.12^**^	−0.13^**^	−0.38^**^	1	
7	Intention to leave	0.26^**^	0.28^**^	0.12^**^	0.08	0.41^**^	−0.61^**^	1

^**^p < 0.01.

As seen in Table 4, There is positive, significant, moderate between workplace violence and WFC relationship (*r* = 0.35, *p* < 0.01), and negative, significant, and weak relationship between workplace violence and job satisfaction (*r* = −0.27, p < 0.01). It was determined that there is a positive, significant, and weak relationship relationship between workplace violence and intention to leave (*r* = 0.26, *p* < 0.01). Correlation analysis also revealed that there is negative, significant, and weak relationship between WFC and job satisfaction (*r* = −0.27, *p* < 0.01), and positive, significant, and weak relationship between WFC and turnover intention (*r* = 0.28, *p* < 0.01).

### 3.4 Hypotheses analysis

In order to evaluate our proposed model, it was conducted a regression-based path analysis employing PROCESS software. This software facilitates the estimation and exploration of interactions and the conditional indirect effects of moderated mediation models (Hayes, 2012). It shares many features with INDIRECT (Preacher and Hayes, 2004) utilizing OLS regression for continuous outcomes to estimate model coefficients and generating direct, indirect, and conditional indirect effects in mediation with single or multiple mediators. Additionally, PROCESS offers tools for investigating two- and three-way interactions and constructs percentile based bootstrap confidence intervals to assess conditional and unconditional indirect effects. By employing bootstrapped confidence intervals, issues related to the asymmetry and non-normal sampling distributions of indirect effects are mitigated (MacKinnon et al., 2004). To test Hypotheses 1 to 4, it was applied Model 4 with 5,000 bootstrap samples and constructed 95% bias-corrected bootstrap confidence intervals for all indirect effects.

*Hypothesis 1 (H1)* proposed that the workplace violence would be negatively associated with job satisfaction of health care workers. The results revealed that workplace violence had a total effect on job satisfaction, *B* = 0.60, *SE* = 0.08, *t* = −6.900, *p* < 0.001, H1 was supported.

*Hypothesis 2 (H2)* proposed that the workplace violence would be positively associated with intention to leave of health care workers. The results revealed that workplace violence had a total effect on intention to leave, *B* = 0.69, *SE* = 0.10, *t* = 6.759, *p* < 0.001. H2 was also supported.

*Hypotheses 3 (H3)* proposed that the work to family (WFC) would mediate the relationship between workplace violence the health care workers face and their job satisfaction. It was observed a significant indirect effect of workplace violence on job satisfaction through WFC, indirect effect = −0.23, *p* < 0.001, 95% CI: -0.332 to −0.152. Given the direct effect of workplace violence remained significant after controlling for WFC, *B* = −0.36, *SE* = 0.08, *t* = −4.180, *p* < 0.001. It was assumed that WFC has a partial mediation effect between workplace violence the health care workers face and their job satisfaction, thus supporting H3.

*Hypotheses 4 (H4)* proposed that the work to family (WFC) would mediate the relationship between workplace violence the health care workers face and their intention to leave. It was observed a significant indirect effect of workplace violence on intention to leave through WFC, indirect effect = 0.31, *p* < 0.001, 95% CI: 0.088–0.152. Given the direct effect of workplace violence remained significant after controlling for WFC, *B* = 0.38, *SE* = 0.10, *t* = 3.752, *p* < 0.001. It was assumed that WFC has a partial mediation effect between workplace violence the health care workers face and their intention to leave. H4 was also supported.

**Figure 2:**
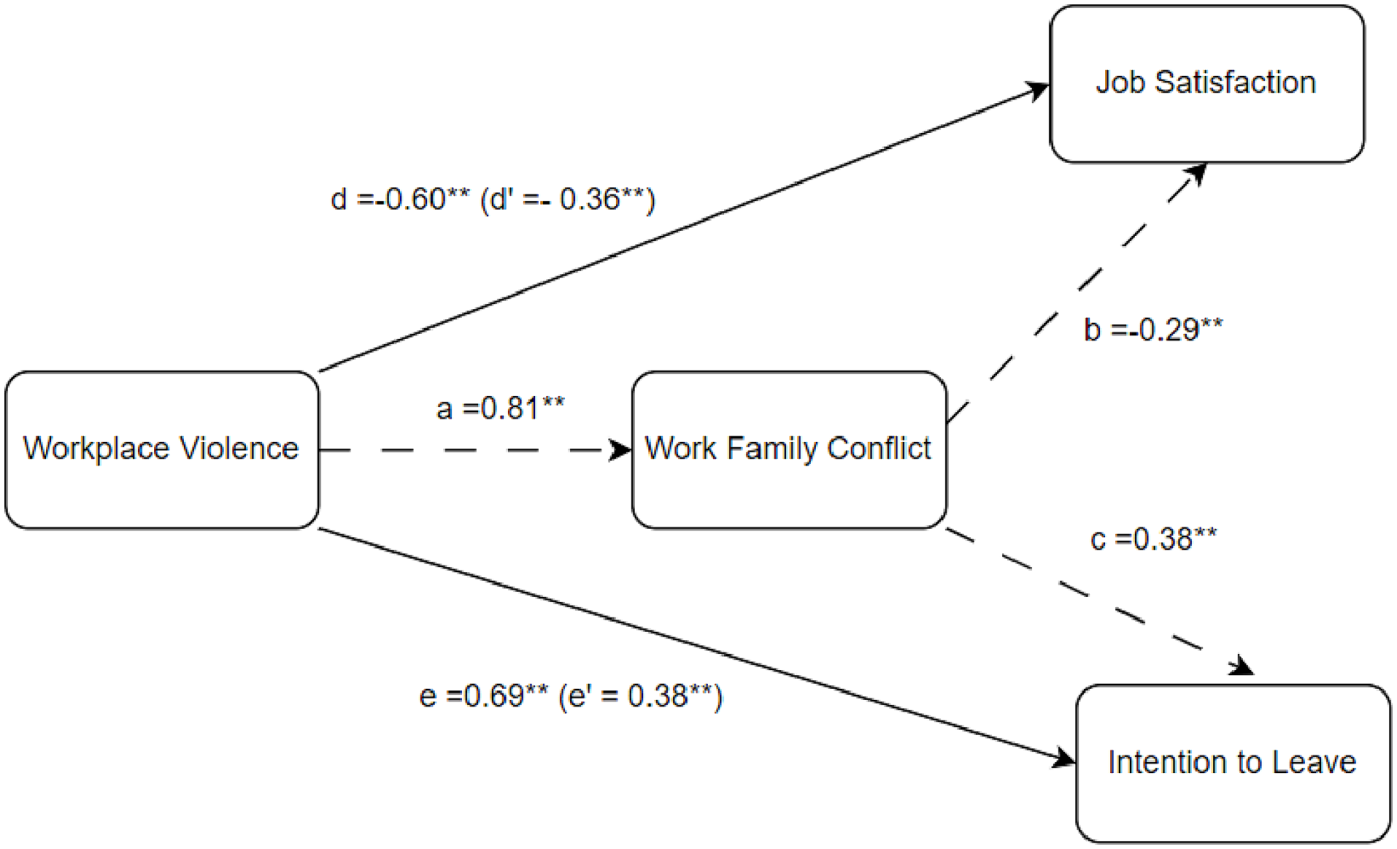
Final model.

The results obtained as a result of the analysis are given in Tables 5, 6 and the final model is given in Figure 2.

**Table 5:** Results of mediation analysis (H1 and H3).

Steps	*B*	*SE*	*t*	*p*
Direct and total effects *R*^2^ = 0.10, *p* < 0.001				
Job satisfaction regressed on workplace violence (*d* path)	−0.60	0.08	−6.90	<0.001
WFC regressed on workplace violence (*a* path)	0.81	0.09	8.42	<0.001
Job satisfaction regressed on WFC, controlling for workplace violence (*b* path)	−0.29	0.03	−8.23	<0.001
Job satisfaction regressed on workplace violence, controlling for WFC (*d’ path*)	−0.36	0.08	−4.18	<0.001

	Unstandardized value	*SE*	LL 95% CI	UL 95% CI	*p*
Bootstrap results for indirect effect					
Effect	−0.23	0.45	−0.332	−0.152	<0.001

List wise *N* = 595. LL, lower limit; CI, confidence interval; UP, upper limit. Bootstrap sample size = 5,000. All predictor variables were mean-centered.

**Table 6:** Results of mediation analysis (H2 and H4).

Steps	*B*	*SE*	*t*	*p*
Direct and total effects *R*^2^ = 0.10, *p* < 0.001				
Intention to leave regressed on workplace violence (*e* path)	0.69	0.10	6.75	<0.001
WFC regressed on workplace violence (*a* path)	0.81	0.09	8.42	<0.001
Intention to leave regressed on WFC, controlling for workplace violence (*c* path)	0.38	0.04	9.47	< 001
Intention to leave on training for workplace violence, controlling for WFC (*e’* path)	0.38	0.10	3.75	<0.001

	Unstandardized value	*SE*	LL 95% CI	UL 95% CI	
Bootstrap results for indirect effect					
Effect	0.31	0.05	0.088	0.157	<0.001

## 4 Discussion

This study aimed to determine how the workplace violence experienced by the employees in the health sector affects their job satisfaction and intention to leave, and also to determine the mediating role of the employees’ WFC between these effects. According to the results of the research, it is seen that the workplace violence levels of the healthcare workers participating in the research are low, WFC is high, and their job satisfaction and intention to leave the job are moderate.

When the results of the correlation analysis between the variables in the research are examined, it is seen that there is a positive, significant, moderate relationship between workplace violence and WFC, and a negative, significant and weak relationship between workplace violence and job satisfaction. There is also a positive, significant, and weak relationship between workplace violence and intention to leave, and a negative, significant, and weak relationship between WFC and job satisfaction. These results are consistent with the results of previous studies in the health sector (Liu et al., 2018; Zhao et al., 2018; Duan et al., 2019).

According to other study results, workplace violence has a significant and negative effect on job satisfaction; a significant and positive effect on the intention to leave, and a significant and positive effect on WFC. It is seen that these results are in parallel with the research results in the literature (Li et al., 2019; Tutan and Öztürk, 2020). In addition, it has been observed that WFC has a significant and negative effect on job satisfaction and a significant and positive effect on the intention to leave. When the mediating role of WFC in workplace violence, job satisfaction, and intention to leave the job was examined, it was determined that WFC had a partial mediator role in the relations between these variables. Similar studies in the literature show that the research results support (Ekici et al., 2017; Li et al., 2019; Huaman et al., 2023).

When we examine the analysis results for the hypotheses we created within the scope of the research, we see that the hypotheses H1, H2, H3, and H4 are confirmed.

Our research reveals that healthcare workers are exposed to different violent behaviors in the workplace environment and its effect on independent variables. These findings indicate that healthcare institutions need to take action to reduce workplace violence and ensure the safety of employees. Among the suggested strategies; Providing comprehensive training for health workers on recognizing, preventing and coping with violence, ensuring that institutional managements in the health sector pay attention to gender balance in employee selection and placement, increasing the effectiveness of the “white code” application used in our country and updating and improving disciplinary procedures, regarding employee health and safety. These include updating the regulations within the scope of workplace violence and strictly implementing the “zero tolerance” policy.

In addition, measures such as reducing the workload of nurses, establishing professional organizations and encouraging interdisciplinary cooperation, increasing education levels and improving working conditions can also contribute to the prevention of violence. Considering the effects of variables such as age, marital status, education level, on-call duty and unit of work on nurses’ exposure to violence, job satisfaction and tendency to quit, institutions need to provide support and working hours appropriate to these factors.

In addition, measures such as reducing the workload of healthcare workers, ensuring that professional organizations are more active in the sector, encouraging interdisciplinary cooperation, increasing education levels and improving working conditions can also contribute to the prevention of workplace violence. Considering the effects of demographic variables such as on-call, unit of work, education level, and age on healthcare workers’ exposure to violence, job satisfaction, WFC, and tendency to quit, institutions need to provide support and a working environment appropriate to these factors.

As a result, the implementation of these recommendations will increase the safety of healthcare workers, contribute to reducing incidents of violence and improving the overall quality of healthcare services.

### 4.1 Limitations and future directions

This research, which aims to determine the job satisfaction, work–family conflict, and intention to leave work of workplace violence experienced by healthcare workers, will contribute to those who will conduct similar research in terms of scientific examination. Although official correspondence was started by considering the units affiliated to the European Union of Public Hospitals affiliated to the Istanbul Provincial Health Directorate during the research planning stage, the practice could only be carried out in the hospitals where permission was given. From this point of view, the results of the study cannot be generalized to the whole health sector and are limited only to the hospitals where the study was applied. Likewise, it is recommended that the study be carried out with more hospitals and health worker clusters in order to generalize the study to the whole health sector in our country.

Finally, it is crucial to identify effective solutions for combating workplace violence. Future research should investigate strategies for reducing or preventing workplace violence and determine best practices in this field.

One of the key strategies should be the implementation of comprehensive workplace violence prevention programs. These programs should include training for healthcare professionals on how to recognize and manage potential violent situations, as well as procedures for reporting violent incidents. Additionally, these programs are required to provide support and resources such as counseling and legal assistance for employees who are victims of workplace violence (Jones, 2021; Rosenbaum et al., 2023).

Another important strategy is to create a safe and respectful working environment (IAHSS, 2021). This can be achieved by implementing strict anti-violence policies, encouraging teamwork and communication among staff, and ensuring adequate staff and resources. In addition, healthcare institutions should try to develop a corporate culture in which all employees feel valued and safe (Somani et al., 2021; Lim et al., 2022).

In addition to these strategies, healthcare organizations should also consider implementing measures to reduce work–family conflict, which has been identified as an important factor contributing to job dissatisfaction and turnover intention among healthcare professionals. This may include offering flexible work schedules, providing child care services, or implementing policies that promote a healthy work-life balance (Oğan and Sercan, 2019; Orellana et al., 2023).

Finally, it is crucial to conduct ongoing research and evaluation to evaluate the effectiveness of these strategies and practices. This will allow healthcare institutions to identify areas for improvement and make necessary adjustments in their efforts to prevent workplace violence (ILO, 2022; The Joint Commission, 2022).

## 5 Conclusion

The results of the above-mentioned analysis indicate that the results of workplace violence experienced by healthcare professionals are not limited to the time spent at work, but also affect the time after work. In this context, concepts related to violence and coping methods with them should be planned as education for health workers in particular and covering their work life, and they should be raised awareness and diversity in this sense.

Determination of job satisfaction and WFC levels of health workers and their intention to leave; will ensure that the institutions have a motivating potential to produce policies in this area and to make the necessary improvement. Identifying the problems in the health sector with the database to be created in this regard. In this sense, it will contribute to raising awareness, thereby establishing social consciousness, and will form a basis for future studies. In addition, it is thought that determining the workplace violence, WFC, and job satisfaction levels of health workers and their intention to leave the job will contribute to the prevention of both material and moral losses, and the productivity and development of the sector.

## Author’s note

This study is a part of the doctoral thesis titled “Antecedents and Consequences of Workplace Violence” conducted by Abdulhamit Tutan within the scope of the Istanbul Sabahattin Zaim University Graduate Education Institute Business Doctorate Program.

## Data availability statement

The raw data supporting the conclusions of this article will be made available by the authors, without undue reservation.

## Ethics statement

The studies involving humans were approved by the İstanbul Sabahattin Zaim University Ethics Commission on 29.04.2022 (E-20292139-050.01.04-27224), and the Istanbul Provincial Health Directorate on 22.09.2022 for use in public hospitals. The studies were conducted in accordance with the local legislation and institutional requirements. The participants provided their written informed consent to participate in this study.

## Author contributions

AT: Writing – review & editing, Writing – original draft, Validation, Software, Investigation, Formal analysis, Data curation, Conceptualization. ÖK: Writing – review & editing, Supervision.
